# Modelling Female Breast Motion During Running: Implications of Breast Support on the Spine

**DOI:** 10.1002/ejsc.12290

**Published:** 2025-04-03

**Authors:** Chris Mills, Timothy A. Exell, Melissa E. A. Jones, Joanna Wakefield‐Scurr

**Affiliations:** ^1^ School of Sport, Health and Exercise Science University of Portsmouth Portsmouth UK

**Keywords:** biomechanics, exercise, modelling, musculoskeletal

## Abstract

During running, it is difficult to control breast motion and change torso motion or vice versa within empirical data collection. This study investigated how different levels of breast support (and consequently breast motion) influence torso motion, breast forces, lumbar and thoracic spinal moments during running, using a computer simulation model. A subject‐specific female full body musculoskeletal model with an articulated thoracolumbar spine and sliding joints between the breasts and torso to enable breast motion was customised for this study. One female (bra size 34DD) had 59 markers attached to anatomical locations and ran over three force platforms at a self‐selected speed (3.15–3.40 m/s) in three breast support conditions (no bra, everyday bra and sports bra). An ‘extreme’ bra condition was simulated during the modelling process by eliminating all breast motion relative to the torso. Two categories of simulations were run, investigating 1) how different breast support garments affect torso motion, breast and spinal moments; and 2) how changes in torso motion affect breast and spinal moments. Key findings suggest that peak lumbar and thoracic spine moments demonstrate changes (> 0.05 Nm/kg) between bra conditions due to changes in running gait kinematics. Additionally, eliminating breast motion relative to the torso, but using the same input running gait kinematics, increased (> 0.05 Nm/kg) lumbar joint moments. Therefore, it is possible that bras aimed at preventing relative motion between the torso and breasts may increase internal loading within the spine.

**Summary**

- A customised subject‐specific female musculoskeletal model was capable of providing a first approximation of changes in spinal moments following simulated changes in breast motion during running.
- Reducing the magnitude of breast motion relative to the torso (∼0.03 m) in this participant, via a more supportive sports bra, caused a reduced torso flexion angle (∼4°) and an increase in peak extension (∼0.15 Nm/kg) spinal joint moments.
- Simulating a theoretical bra product that could eliminate all breast motion relative to the torso (100% bounce reduction), but uses identical experimental gait kinematics increased (0.04 to 0.10 Nm/kg) peak lumbar extension moments.
- Improvements in bra design that aim to maximise bounce reduction may increase internal loading on the spine and hence possible back pain during running.

## 1 Introduction

Due to their lack of intrinsic support during dynamic activity, breasts deform (McGhee and Steele 2020; Risius et al. 2015) and move relative to the torso (Mills et al. 2014). Breast support garments, such as bras, provide additional extrinsic support to reduce breast movement and subsequently minimise exercise‐induced breast pain (J. White et al. 2015). In both research and commercial activity, bras are commonly categorised as high support, which usually refers to sports bras, or lower support, which can refer to everyday/fashion bras (McGhee et al. 2013; J. L. White et al. 2009). All bras afford different amounts of breast motion reduction depending upon their design and construction (Wang et al. 2017).

Many bra manufacturers place emphasis within their marketing on ‘bounce reduction’ with greater reductions in breast motion indicating improvements in design and bra performance (Bunchanan and Joshi 2023; Oluban 2023). Although reducing breast motion has been shown to reduce breast pain (Brown et al. 2014; McGhee and Steele 2020), it may be that bra designs with greater breast motion reduction have an ‘unseen’ consequence on the musculoskeletal system. Soft tissue motion within other parts of the body, such as the thigh or shank, has been reported as an important factor in reducing internal joint loading (Pain and Challis 2006; Gittoes et al. 2006). The magnitude of soft tissue motion of the breasts may alter the joint moments within the spine, and hence reducing soft tissue breast motion (improved bounce reduction within a bra) may in fact increase spinal moments.

Spinal loading plays an important role in back pain (Actis et al. 2018) and a difference of 0.05 Nm/kg in lumbar spine moments has been reported between male participants with and without back pain (Hasegawa et al. 2018). Postural changes at the thoracic spine, due to potentially heavy loads from the breasts on the anterior chest, have also been a proposed causal mechanism for nonspecific back pain (McGhee et al. 2018). Nonspecific back pain has been reported to be greatest in the lumbar and thoracic regions, with breast support garments that reduce the nipple‐to‐sternal notch distance relieving nonspecific back pain (Haworth et al. 2023). Finally, Leme et al. (2020) stated that if the appropriate breast support is not used, women may perform adaptations throughout the body to relieve the discomfort of breast movement when not supported. This suggests that the magnitude of breast support afforded by bras may be linked to changes in vertebrae alignment, and hence potentially changes in spinal moments of greater than 0.05 Nm/kg may provide an indication of increased risk of potential back pain (Hasegawa et al. 2018).

The musculoskeletal modelling approach has been utilised to understand how occupational load carriage tasks alter spinal load (Kim and Zhang 2017) and to estimate internal spinal loading, providing an insight into the underpinning mechanism causing back pain. However, previous female musculoskeletal models have either incorporated breast mass within the torso segment or as fixed segments on the anterior of the torso (Morino and Takahashi 2017), neglecting relative motion between the torso and breasts.

As previous research reported that soft tissue motion reduced internal joint loading, it is hypothesised that increases in breast motion relative to the torso will decrease the internal loading within the spine, during an activity like running. However, the magnitude of breast motion may also affect torso motion (Fong et al. 2022), leading to adjustments in torso or full body mechanics to compensate for changes in forces elsewhere. During running, it is difficult to control breast motion and change torso motion or vice versa within empirical data collection; however, this novel application of computer modelling provides a unique way to assess breast motion changes and their effects on torso motion and spine moments.

Therefore, this study aims to investigate how breast motion changes effect torso motion, breast forces and lumbar and thoracic spinal joint moments. Subsequently, two objectives were created: Objective 1, to understand how different simulated breast support garments (sports bra, everyday bra and no bra) affect torso motion, breast and spinal joint moments during running. Objective 2, to simulate a bra with 100% bounce reduction to understand how completely constraining breast motion relative to the torso, when running, would change breast forces and spinal joint moments.

## 2 Materials and Methods

**FIGURE 1:**
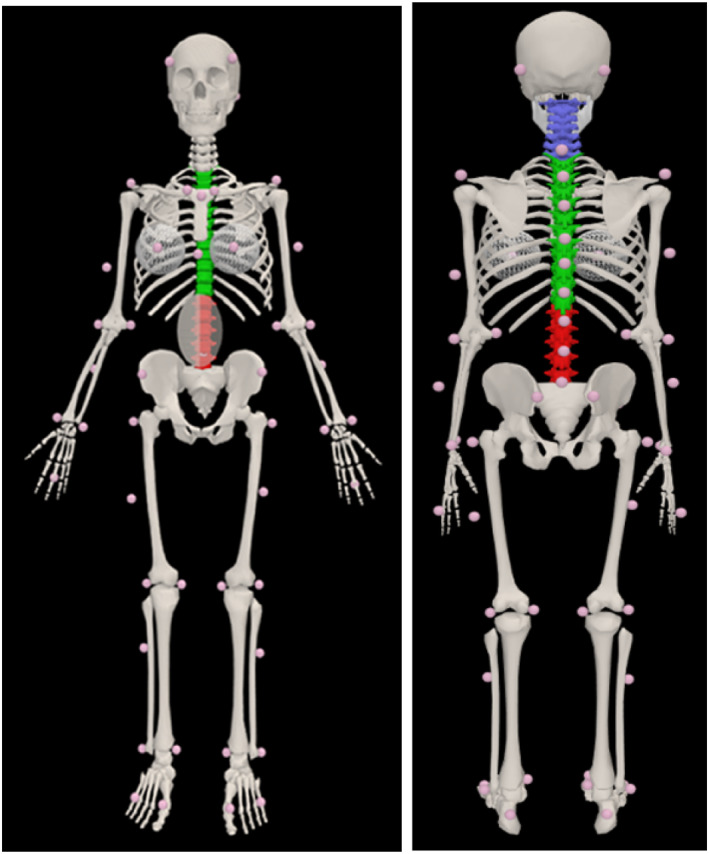
Customised marker locations on participants. Lumbar spine markers located at L1, L3 and L5 and thoracic markers located at T1, T3, T5, T7, T9 and T11.

Following institutional ethical approval, one female (74.6 kg, 1.79 m, 26 years, UK bra size 34DD) provided written informed consent to participate. One participant was recruited for this study and one trial in each condition used, similar to previous modelling research (Mills et al. 2008; Pain and Challis 2006; Masters and Challis 2022), as the purpose of this paper was to understand the underpinning mechanics of how simulated breast movement reduction effects the spine rather than the variability of an individual's performance. A customised marker set of 57 reflective markers was attached to anatomical locations (Figure 1), with a handheld ultrasound machine (Sonosite Edge, USA) used to guide spine marker placement. One additional marker was placed on each nipple (over the bra, where appropriate). Three force platforms (9281E, Kistler, Switzerland; 1000 Hz) and a 19‐camera motion capture system (Qualisys, Sweden; 250 Hz) were synchronised for data collection. Left and right breast boundaries were identified using the folding line method (H. Lee et al. 2004) and the most superior, inferior, medial and lateral boundary positions marked using a surgical pen for breast volume and mass estimation. The participant completed a running trial in three bra conditions: bare breasted (no bra; NB), wearing a nonpadded, underwired everyday bra (EB) (Marks & Spencer; made from 88% Polyamide and 22% elastane Lycra) and a sports bra (SB) (Triumph, Triaction; made from 30% polyamid, 25% elastan, 25% cotton, 20% polyester) and had their bra size assessed by a trained bra fitter using the best‐fit criteria (J. White and Scurr 2012). The participant stood statically for 15 s while a handheld 3D surface scanner (Artec Eva) recorded torso and breast geometry in each bra condition to estimate breast volume, mass and centre of mass location. Following a gentle warm‐up and familiarisation, the participant stood statically for 5 s and then performed separate running trials at a self‐selected speed (3.15–3.40 m/s), in each bra condition (to maximise ecological validity), while kinematic and kinetic data were collected.

Surface breast scans were processed in Artec Studio 17 Professional (Artec3D, Luxembourg) using the marked breast boundary in the no‐bra condition. Each breast was extracted from the torso and the posterior of the breast was flat filled and the volume calculated using the software. Breast mass was estimated using the breast volume and breast density of 945 kg/m^3^ (Sanchez et al. 2017), giving a mass of 0.747 kg (right) and 0.754 kg (left). Second, for each bra condition (no bra, everyday bra, sports bra), the breast centre of the mass position was calculated using the static breast 3D surface scan and assuming each breast was a geometric solid hemisphere (Haddox et al. 2020). The centre of mass was calculated using *Y* = 3 R/8, where ‘Y’ was the distance to the centre of mass and ‘R’ was the radius of the hemisphere. The radius was calculated from the nipple marker (breast apex) to the breast boundary. The centre of mass was at a distance Y anteriorly from the breast base towards the breast apex. A virtual breast centre of the mass marker was created in all static and dynamic trials within the Qualisys Track Manager software. All kinematic and kinetic data were processed and exported via a customised MATLAB script for importing into OpenSim (Simtk.org).

A validated female full‐body model (Burkhart et al. 2020) with a fully articulated thoracolumbar spine (T1 through L5) and 3 rotational degrees of freedom at each intervertebral joint was selected for customisation for this study. The female model was modified to include two breast segments positioned on the anterior of the torso. Previous literature has defined the motion of the breast in three dimensions (Scurr et al. 2011); therefore, each breast segment was represented by a point mass and attached via a three degrees‐of‐freedom sliding joint to the torso allowing the translation of the breast segment relative to the torso segment in three planes (anterior/posterior, medial/lateral and superior/inferior). This is a tracking‐based (data‐driven) inverse dynamics solution and the viscoelastic behaviour of the breast tissue is inherently included in the experimental kinematic data. The initial position of each breast segment was defined by the centre of the mass position of the breast during standing. The standard OpenSim workflow (Akhavanfar et al. 2022) was followed to ensure the generic female model (Burkhart et al. 2020), scaled to the participant based on marker data from the static trial (model to experimental marker RMS < 1 cm, maximum error < 2 cm) and was within recommended thresholds (Hicks 2019). The breasts were not included in the scaling process and their mass was included within the torso segment. After successful completion of the scaling process, the mass of each breast was subtracted from the sum of the lumbar and thoracic spine, ribs, sternum, clavicular and abdominal segment (torso) masses (24.04 kg) and added to the relevant breast segment; therefore, the whole‐body mass remained unchanged. The Inverse Kinematics Tool, in OpenSim, was used to find the values for the generalised coordinates (joint angles and positions) in the model that best matched the experimental kinematics using a weighted least squares approach, whose solution aimed to minimise both marker and coordinate errors. The resulting kinematics were filtered with a low pass Butterworth filter and a cut‐off frequency of 5 Hz (Rácz and Kiss 2021). Subsequently, the inverse kinematics solutions were combined with the ground reaction force data within the Inverse Dynamics Tool for each bra condition in order to calculate the spinal joint moments during the running trials. This protocol enabled us to understand how different bras affect torso motion and spinal moments (Objective 1).

To address Objective 2, a hypothetical bra condition (extreme bra) was created with the breast segments locked in their static trial position within the OpenSim software. The inverse dynamics were re‐run using the running gait kinematics from the other bra conditions (no bra, everyday bra and sports bra). This simulated an ‘extreme’ sports bra condition in which all relative motion between the breast and torso segments was eliminated (100% bounce reduction). This allowed any changes in running gait kinematics caused by previous bra conditions to be examined independently of the breast support provided by the bra for the participant in this study.

The local coordinate system for the torso was defined by markers on the sternal notch, xiphoid process, seventh cervical vertebra and eighth thoracic vertebra (Wu et al. 2005) and used to calculate torso flexion/extension, lateral bend and axial rotation within the global coordinate system for each breast support condition. Nipple markers were used to calculate the range of motion relative to the torso segment (Mills et al. 2016) during the gait cycle of each breast support condition to ensure the selected trial was indicative of typical published nipple motion data (Mills et al. 2016; J. L. White et al. 2009; Scurr et al. 2010) and hence suitable for subsequent comparison between breast support conditions. The nipple range of motion (No bra = 0.057 m superior‐inferior (SI); 0.034 m mediolateral (ML); 0.035 m anterior‐posterior (AP). Everyday bra = 0.022 m (SI); 0.014 m (ML); 0.008 m (AP). Sports bra = 0.022 m (SI); 0.008 m (ML); 0.004 (AP)). Subsequently, the breast centre of the mass position and breast joint force (the force applied by the breasts on the torso at the sliding joint) was calculated in the torso local coordinate system in three directions (anterior‐posterior, medial‐lateral and superior‐inferior). For each bra condition, the net joint moment of each lumbar (L1–L5) and thoracic spine joints (T1–T12) were averaged to give single lumbar and thoracic moment values at each time point (Raabe and Chaudhari 2016). Time histories were normalised and key events within the gait cycle were identified using the vertical ground reaction forces (threshold > 10 N). All spinal joint moment data were normalised to the participant's mass (74.6 kg).

## 3 Results

### 3.1 Objective 1

**FIGURE 2:**
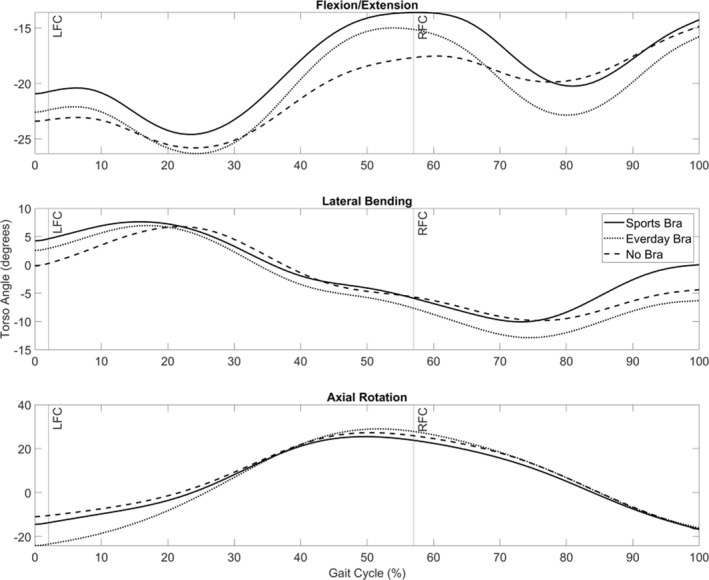
Torso angle during the running gait cycle (LFC = left foot contact; RFC = right foot gait contact) (flexion (−), extension (+); left bend (−), right bend (+); clockwise rotation (−), anticlockwise rotation (+)).

The participant's self‐selected horizontal velocity was 3.40 m/s in no bra, 3.27 m/s in the everyday bra and 3.14 m/s in the sports bra. The torso remained in flexion throughout the trials (∼11° range of motion), with symmetrical axial rotation and slightly greater (∼4°) left bend of the torso (Figure 2). Figure 2 suggests that for the participant in this study, the bra condition affected the orientation of the torso, with a more upright torso position in the sports bra condition. However, the bra condition did not alter the torso range of motion during the running gait cycle.

**FIGURE 3:**
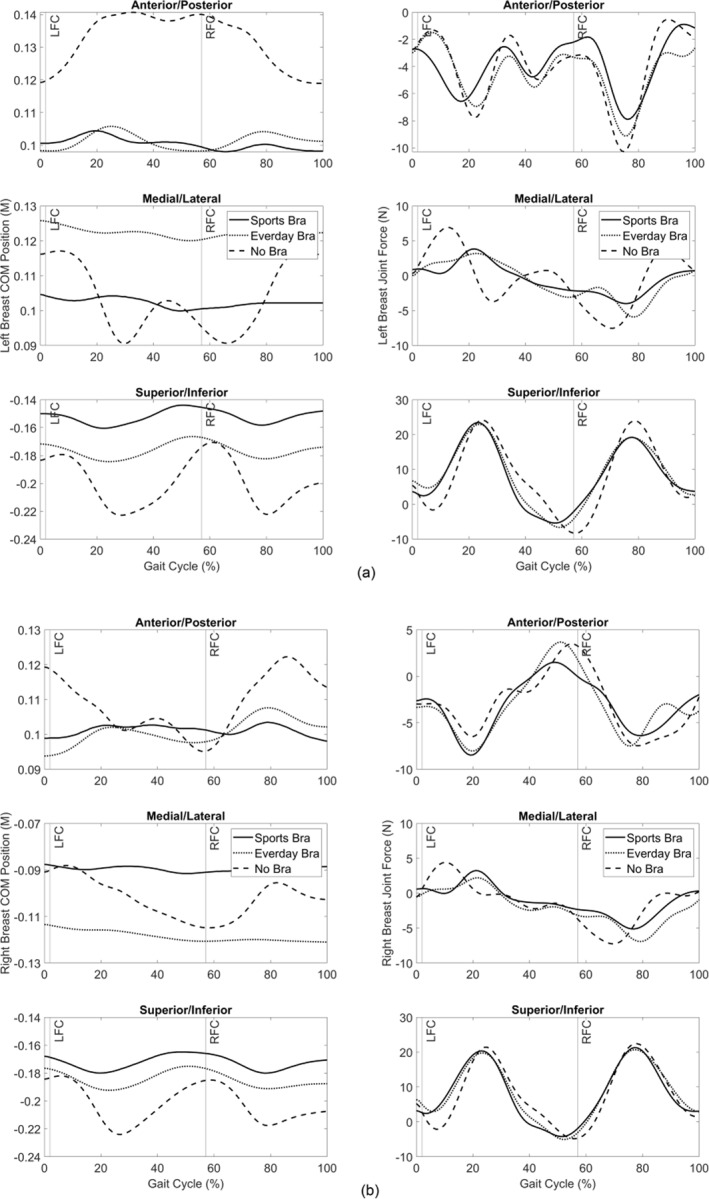
(a) Left breast centre of mass position (left figures) and breast joint force (right figures). (b) Right breast centre of mass position (left figures) and breast joint force (right figures) during the running gait cycle in each bra condition. (LFC = left foot contact; RFC = right foot gait contact).

The greatest range of motion of the breast centre of mass (∼0.052 m) occurred during no‐bra running in the superior‐inferior direction (Figure 3). Following decreases in the breast centre of mass motion, the magnitude of peak breast joint force also decreased (up to 4 N) between bra conditions. There were small changes in the timing of peak breast joint forces (Figure 3). Peak lumbar and thoracic spine moments showed differences (> 0.05 Nm/kg) between bra conditions in most planes (Table 1), with a trend of increased lumbar extensor moments with increased breast support.

**TABLE 1:** Peak lumbar and thoracic joint moments during the running gait cycle in three bra conditions (NB = no bra; EB = everyday bra; SB = sports bra). (Flexion (Flex), extension (Ext); left bend (LB), right bend (RB); clockwise rotation (CW), anticlockwise rotation (ACW)).

Bra condition	Peak lumbar joint moment (Nm/kg)			Peak thoracic joint moment (Nm/kg)		
	Flex (−) Ext (+)	LB (−) RB (+)	CW(−) ACW(+)	Flex (−) Ext (+)	LB (−) RB (+)	CW(−) ACW(+)
SB	−0.07^a^ +1.32^b^	−0.59^b^ +0.60^a^, ^b^	−0.71^b^ +0.71	−0.08 +0.44	−0.41^b^ +0.42^b^	−0.42^a^, ^b^ +0.39^a^, ^b^
EB	−0.12^a^, ^c^ +1.28^b^, ^c^	−0.60^c^ +0.81^a^, ^c^	−0.74^c^ +0.73	−0.11 +0.43	−0.38^c^ +0.47^c^	−0.34^a^, ^c^ +0.47^a^
NB	−0.05^c^ +1.17^b^, ^c^	−0.54^b^, ^c^ +0.86^b^, ^c^	−0.95^a^, ^c^ +0.72	−0.08 +0.42	−0.54^b^, ^c^ +0.61^b^, ^c^	−0.52^b^, ^c^ +0.50^b^

^a^ difference in SB & EB > 0.05 Nm/kg.

^b^ difference in SB & NB > 0.05 Nm/kg.

^c^ difference in EB & NB > 0.05 Nm/kg.

### 3.2 Objective 2

**FIGURE 4:**
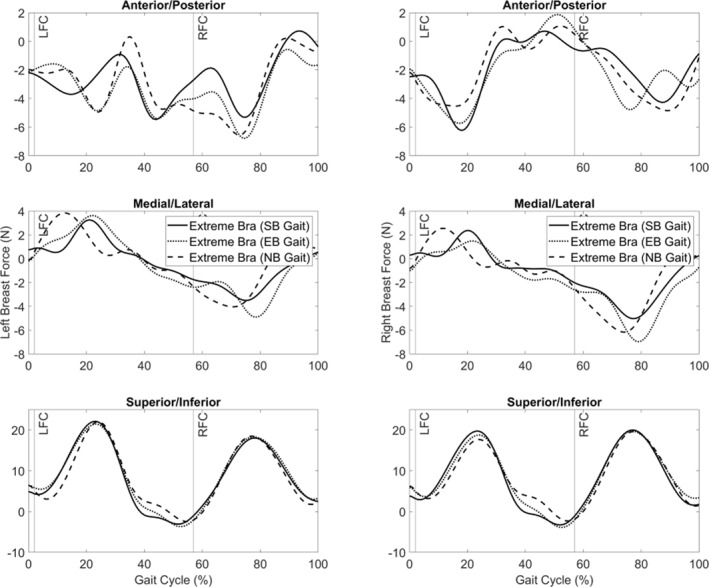
Left and right breast joint force during the running gait cycle (LFC = left foot contact; RFC = right foot gait contact) in the extreme bra condition using the gait kinematics from the three breast support conditions. (NB = no‐bra gait kinematics; EB = everyday bra gait kinematics; SB = sports bra gait kinematics).

Despite relative breast motion being zero, a force is still applied at the breast joints during running. There were minimal changes (∼2 N) in peak breast joint force between the three bra gait kinematics when breast motion was zero (Figure 4). A phase delay of ∼10% of the gait cycle was apparent for breast joint force in the medial‐lateral direction within the sports bra and everyday bra running gait kinematics compared to the no bra.

Constraining breast motion to zero (extreme bra) caused peak lumbar and thoracic spine moments to change by more than 0.05 Nm/kg, depending on input gait kinematics (e.g., gait kinematics from the no bra, everyday bra or sports bra trials) (Table 2). The greatest magnitude of joint moments occurred during lumbar extension, with a 0.09 Nm/kg (7%) increase, to 1.36 Nm/kg, in extensor moments when running with no bra compared to sports bra gait kinematics. However, the greatest change was seen in the gait kinematics from the everyday bra trial reducing clockwise loading by 32% (0.36 Nm/kg reduction) compared to the no‐bra gait kinematics; furthermore, there was a 0.28‐Nm/kg (32%) reduction in the right bending from no‐bra to sport bra gait kinematics.

**TABLE 2:** Peak lumbar and thoracic joint moments during the running gait cycle for the extreme bra condition, when the motion of the breast centre of mass was fixed at zero (relative to the torso) and the model used the gait kinematics generated during running in each of the bra conditions (NB = no bra gait kinematics; EB = everyday bra gait kinematics; SB = sports bra gait kinematics). (Flexion (Flex), extension (Ext); left bend (LB), right bend (RB); clockwise rotation (CW), anticlockwise rotation (ACW)).

Bra condition	Peak lumbar joint moment (Nm/kg)			Peak thoracic joint moment (Nm/kg)		
	Flex (−) Ext (+)	LB (−) RB (+)	CW (−) ACW (+)	Flex (−) Ext (+)	LB (−) RB (+)	CW (−) ACW(+)
SB	−0.21^a^, ^b^ +1.36^b^	−0.59^b^ +0.59^a^, ^b^	−0.92^a^, ^b^ +0.69^a^	−0.13 +0.48	−0.40 +0.44^b^	−0.52^a^, ^b^ +0.38^a,b^
EB	−0.13^a^ +1.33^c^	−0.56 +0.80^a^, ^c^	−0.78^a^, ^c^ +0.74^a^	−0.13 +0.46	−0.38^c^ +0.48^c^	−0.35^a^, ^c^ +0.47^a^, ^c^
NB	−0.11^b^ +1.27^b^, ^c^	−0.54^b^ +0.87^b^, ^c^	−1.14^b^, ^c^ +0.71	−0.12 +0.49	−0.43^c^ +0.59^b^, ^c^	−0.62^b^, ^c^ +0.59^b^, ^c^

^a^ difference in SB & EB > 0.05 Nm/kg.

^b^ difference in SB & NB > 0.05 Nm/kg.

^c^ difference in EB & NB > 0.05 Nm/kg.

**FIGURE 5:**
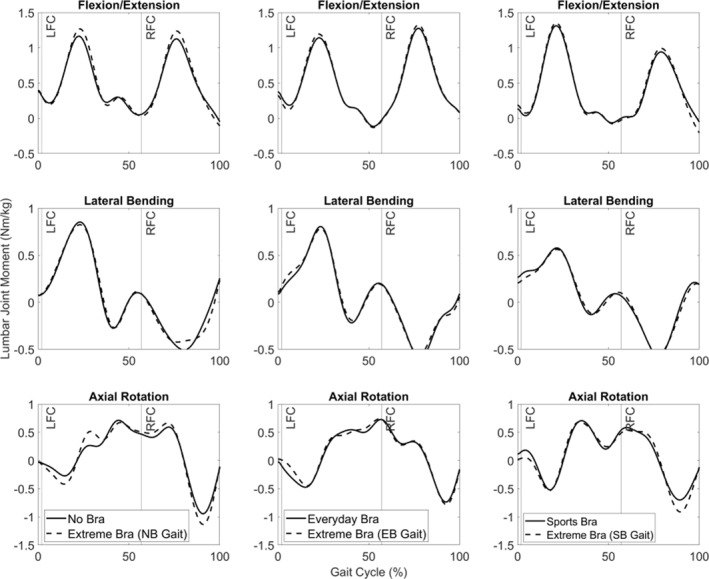
Average lumbar joint moments during the running gait cycle (LFC = left foot contact; RFC = right foot gait contact) in all bra and gait conditions. Flexion (−), extension (+); left bend (−), right bend (+); clockwise rotation (−), anticlockwise rotation (+) NB = no bra gait kinematics; EB = everyday bra gait kinematics; SB = sports bra gait kinematics.

**FIGURE 6:**
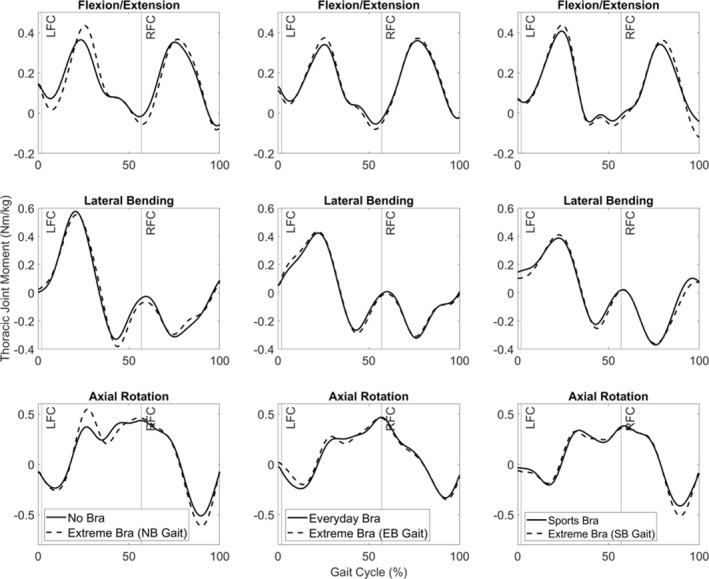
Average thoracic joint moments during the running gait cycle (LFC = left foot contact; RFC = right foot gait contact) in all bra and gait conditions. Flexion (−), extension (+); left bend (−), right bend (+); clockwise rotation (−), anticlockwise rotation (+). NB = no bra gait kinematics; EB = everyday bra gait kinematics; SB = sports bra gait kinematics.

When examining lumbar joint moment time histories in the extreme bra trial, there were minimal differences in lateral bending; axial rotation showed both increases and decreases in lumbar moments time histories when compared to their corresponding gait kinematics (Figure 5). However, extension lumbar joint moments tended to be greater in the extreme bra conditions and the no‐bra gait kinematics exceeded 0.05 Nm/kg (Figure 5). When examining the thoracic joint moment time histories, the magnitudes were lower than the lumbar spine (Figures 5 and 6); however, similar patterns to the lumbar spine were evident with some joint moments exceeding 0.05 Nm/kg in the extreme bra when compared to their corresponding gait kinematics.

## 4 Discussion

This study used a subject‐specific female musculoskeletal model to investigate how different bras influence torso motion, breast joint forces and lumbar and thoracic spinal moments during running. Key findings suggest that reducing the magnitude of breast motion relative to the torso (∼0.03 m), via a more supportive sports bra, is associated with a reduction in the torso flexion angle (∼4°) and an increase in peak extension (∼0.15 Nm/kg) spinal joint moments, compared to running in no bra, for the participant modelled in this study. Furthermore, when simulating an ‘extreme’ bra that eliminated breast motion relative to the torso, but used identical experimental gait kinematics from each bra condition, the ‘extreme’ bra condition increased (0.04 to 0.10 Nm/kg) peak lumbar extension moments. Although the bra industry may strive towards maximising bounce reduction, this novel method of modelling breast motion relative to the torso has demonstrated that achieving 100% bounce reduction will not enable any energy dissipation and hence may increase internal loading on the spine and possible risk of back pain.

The female musculoskeletal computer model developed for this study enabled the authors to understand how different breast support garments (bras) affected torso motion, breast joint forces and spinal moments. The breast support conditions (no bra, everyday bra and sports bra) were associated with changes in the self‐selected horizontal running velocity, the magnitude and timing of breast centre of mass position, torso motion and breast joint forces during running for the participant and trials in this study. The self‐selected horizontal running velocity was 0.26 m/s lower in the sports bra compared to the no‐bra condition. Furthermore, the greatest breast range of motion occurred within the no‐bra condition and in the superior‐inferior direction, similar to previous studies (Scurr et al. 2010). Both the sports bra and everyday bra attenuated superior‐inferior breast motion similarly; however, the sports bra lifted the breast more superiorly relative to the torso (by ∼0.01 m). The sports bra was more successful at reducing breast motion in other directions than the everyday bra when compared with no bra. A different horizontal running velocity in each bra condition may have contributed to the reduction in the magnitude of torso flexion by ∼4° in the sports bra compared to the no bra, suggesting a slower but more upright running technique. The torso range of motion remained similar across bra conditions.

The force applied by the breasts on the torso at the sliding joint (breast joint force) also reflected similar changes to the breast motion, supporting previous evidence that a combination of design features and viscoelastic properties of the fabric materials used can alter breast motion (Zhou et al. 2013; C.‐w. Lee et al. 2022). Finally, peak lumbar and thoracic spine moments showed (> 0.05 Nm/kg) differences between bra conditions, possibly indicating a change in the potential for the participant to experience back pain (Hasegawa et al. 2018). Running in no bra changed the gait kinematics, reduced peak lumbar extensor moments, increased peak right bending and clockwise rotation moments compared to the sports bra condition. Furthermore, running in no bra increased left and right bending moments and clockwise and anticlockwise rotation moments within the thoracic region when compared to the sports bra condition. Thus, running while wearing a sports bra may increase the risk of lumbar back pain (indicated by an increased extension moment > 0.05 Nm/kg; Hasegawa et al. 2018), but it may also help reduce thoracic back pain. The results associated with Objective 1 suggest that the ‘level’ of breast support (i.e., amount of breast movement reduction relative to the torso) does impact the magnitude of breast force, torso position and lumbar and thoracic spinal moments for the participant in this study.

The bra industry increasingly markets ‘bounce reduction’ or reduction in relative breast motion as a measure of bra performance. The extreme bra condition was created to simulate a theoretical product that could eliminate all relative breast motion while maintaining gait kinematics from the other experimental bra conditions (Objective 2). The results suggest that the change in gait kinematics associated with the different bra conditions (no bra, everyday bra and sports bra) has a similar impact on the magnitude of spinal moments as the breast motion reduction afforded by the bras. For example, the difference in peak lumbar extension moments between bra conditions was 0.15 Nm/kg; however, the difference due to the extreme bra (while maintaining matched bra gait kinematics) was 0.09 Nm/kg. Therefore, if the participant in this study ran with the gait kinematics observed in the sports bra trial while wearing a hypothetically more supportive ‘extreme’ bra, the limited energy dissipation would likely lead to increased spinal loading, particularly in flexion, extension, and right bending. It is suggested that the participant in this study may have used a compensatory mechanism whereby gait kinematics were altered as breast kinematics changed due to the bra condition in order to maintain a similar level of spinal moments. This, in combination with theoretical mechanics, suggests that bra industries that strive for greater bounce reduction in their products may need to consider the ‘unseen’ consequence on the musculoskeletal system such as compensatory mechanisms or changes in spinal loading.

This study offers a unique new insight into the effect of breast support on spinal moments using a musculoskeletal modelling approach, conflicting with the common assumption that breast movement reduction is always a positive outcome in sports bra design. Although this study investigated three bra conditions and a further simulated ‘extreme’ bra condition, future research could look to find the optimal combination of breast motion and timing (bounce reduction) that minimises spinal loading to inform future bra design. Biomechanical or engineering‐related modelling research that utilises females could benefit from including moveable breast segments into models to ensure improved realism of female torso characteristics that could impact equipment design or estimating of internal loading within female injury risk prediction.

Although the results of this study demonstrate differences in spinal moments between simulated changes in breast motion, the limitations associated with this modelling approach are important to discuss. The actual data presented may vary given the individual single subject nature of the model and single trial approach used, and thus aspects such as the self‐selected individual gait kinematics of participants in different bras may influence whole body kinematics (Taylor M et al. 2020; Fong et al. 2022) and the exact spinal moments reported; however, the general principles can be inferred (Yeadon and Pain 2023). The proposed threshold of 0.05 Nm/kg (Hasegawa et al. 2018) as an indicator of possible changes in back pain must be considered given this was reported for male participants and the lumbar spine. Therefore, although this may provide an indicator, any conclusion regarding bras and back pain must be considered with caution and could be investigated in future work with symptomatic patients while standing, walking and running. The breast kinematics were captured using a marker placed on the bra; although careful bra fit criteria were followed (J. White and Scurr 2012), it may be possible for relative motion between the breast tissue and bra to occur, altering the magnitude of actual breast motion. This tracking‐based (data‐driven) inverse dynamics solution incorporated the basic gross level viscoelastic behaviour of the breast tissue inherently in the experimental kinematic data; however, a future forward dynamics model would enable the manipulation of viscoelastic soft tissue properties to determine how they affect soft tissue motion. Finally, although this model did not account for musculature and the resulting muscle contraction forces on spinal joint compression and shear, the model utilised the same input gait kinematics to provide an initial insight into the underpinning mechanisms of how changes in breast motion could affect spinal moments during running.

## 5 Conclusion

Although caution must be applied when interpreting the magnitude of differences in spinal moments between the simulated changes in breast motion, there is evidence to suggest that an individualised subject‐specific female musculoskeletal model was capable of providing a first approximation of changes in spinal moments following simulated changes in breast motion. Furthermore, it is possible that a bra that achieves 100% bounce reduction may increase extensor loading within the spine.

## Conflicts of Interest

The authors declare no conflicts of interest.

## Data Availability

The data that support the findings of this study are available from the corresponding author upon reasonable request.
